# Trigger mechanism for the abrupt loss of energetic ions in magnetically confined plasmas

**DOI:** 10.1038/s41598-018-21128-z

**Published:** 2018-02-12

**Authors:** K. Ida, T. Kobayashi, M. Yoshinuma, T. Akiyama, T. Tokuzawa, H. Tsuchiya, K. Itoh, S.-I. Itoh

**Affiliations:** 1 National Institute for Fusion Science, National Institutes of Natural Sciences, Toki, 509–5292 Japan; 2 SOKENDAI (The Graduate University for Advanced Studies), 322-6 Oroshi, Toki, Gifu 509–5292 Japan; 3 Institute of Science and Technology Research, Chubu University, Kasugai, 487–8501 Japan; 4 Research Institute for Applied Mechanics, Kyushu University, Kasuga, 816–8580 Japan

## Abstract

Interaction between a quasi-stable stationary MHD mode and a tongue-shaped deformation is observed in the toroidal plasma with energetic particle driven MHD bursts. The quasi-stable stationary 1/1 MHD mode with interchange parity appears near the resonant rational surface of *q* = 1 between MHD bursts. The tongue-shaped deformation rapidly appears at the non-resonant non-rational surface as a localized large plasma displacement and then collapses (tongue event). It curbs the stationary 1/1 MHD mode and then triggers the collapse of energetic particle and magnetic field reconnection. The rotating 1/1 MHD mode with tearing parity at the *q* = 1 resonant surface, namely, the MHD burst, is excited after the tongue event.

## Introduction

Abrupt loss of plasma energy due to MHD activity has been commonly observed and various collapse events have been reported in toroidal plasmas^1^. One of the mysteries of the abrupt collapse event is why the MHD mode is stable although the critical parameters seem to exceed the linear stability limit. The concept of fragile stability thus has been proposed^2^. In order to explain the transition from fragile stability to an actual unstable state, an abrupt event which triggers the collapse is necessary. This is recognized as a trigger problem and is commonly observed in magnetized plasma in tokamak plasmas^3,4^ and solar flares^5^. In the solar flare, the reversed magnetic shear and polarity inversion are proposed as a mechanism causing the onset of solar eruptions^5,6^. In tokamaks, the edge-localized mode (ELM), especially the so-called type I ELM, is one of the MHD instabilities causing the loss of plasma kinetic energy as an abrupt collapse event. A narrow fingerlike perturbation growing radially from a poloidally elongated filament initiates the collapse^7,8^.

In helical plasmas, there are various energetic particle driven MHD instabilities observed^9,10^. The loss of energetic particles are also observed associated with the energetic particle driven MHD bursts^11,12^. The bursting *m*/*n* = 1/1 instability, namely, the energetic ion driven resistive interchange (EIC) mode, in the plasma peripheral region by resonant interaction between helically trapped energetic ions and the resistive interchange mode was reported^13^. In order to clarify the causality between the MHD burst and the loss of energetic ions, precise measurement of the timing of the energetic ion loss is necessary. Therefore, the intensity of RF radiation probes is used as a timing indicator for the energetic ion loss from the plasma^14^ in this work because the RF probe has high time resolution and is sensitive to the high frequency RF signals excited by the loss of energetic ions at the plasma edge^15,16^. Although there is a quasi-stable 1/1 MHD mode observed between bursts, it was also not clear how this MHD burst is triggered. Recently, a tongue-shaped deformation^17,18^ is observed just before the MHD bursts^19^. This is considered to be a candidate for the trigger mechanism causing the MHD bursts. In this discharge, the beta value (a ratio of plasma pressure to magnetic pressure) is low at 0.4% and the tongue-shaped deformation is not caused by the gradient of bulk pressure but rather by the gradient of an energetic ion population injected by neutral beams. Therefore, the relation between the collapse of energetic ions and the tongue-shaped deformation is essential. In order to investigate the trigger mechanism, the study of mode structure and plasma displacement with high time resolution (∼10 $\mu$s) which is much shorter than one period of the MHD mode is necessary, because the growth rate of the instability triggering the MHD bursts (∼10^4^s^-1^) is larger than the 1/1 MHD mode frequency (5–8 kHz).

Here we report the interaction between the quasi-stable MHD mode and tongue-shaped deformation. The tongue-shaped deformation curbs the stationary 1/1 MHD mode but triggers the collapse of energetic particles and the change of parity of the 1/1 MHD mode due to the magnetic field reconnection at the rational surface. The causality relation between the MHD burst and the collapse of energetic ions is also discussed.

## Results

### Observation of MHD Burst

**Figure 1:**
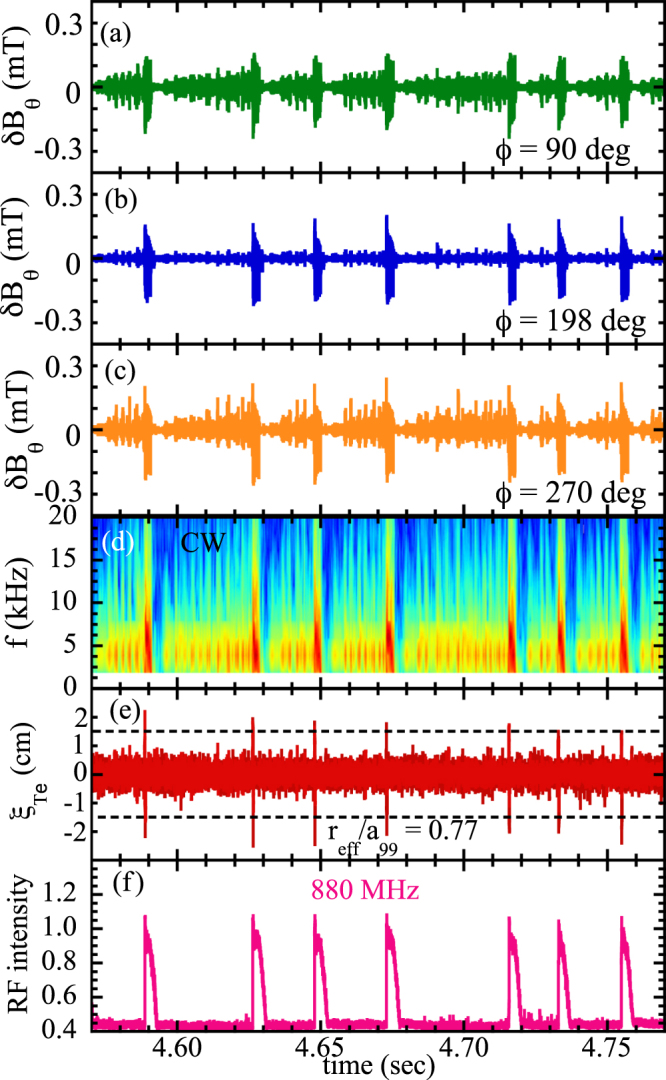
Time evolution of poloidal magnetic field perturbation, $\delta {B}_{\theta }$, at toroidal angles *ϕ* of (**a**) 90°, (**b**) 198°, and (**c**) 270° in toroidal array, (**d**) contour of 1/1 mode amplitude of $\delta {B}_{\theta }$, (**e**) displacement of the contour of electron temperature at ${r}_{{\rm{e}}ff}/{a}_{99}$ = 0.77, and (**f**) RF intensity at 880 MHz measured with RF radiation probe) in shot #116190.

The Large Helical Device (LHD) is a Heliotron-type device equipped with three tangential neutral beams with a beam energy of 160–180 keV and two perpendicular beams with a beam energy of 40–50 keV. When the two perpendicular beams are injected into the plasma with relatively low density of 1–2 × 10^19^ m^−3^, the MHD bursts appear to be associated with the enhancement of high frequency RF signals. Figure 1 shows the time evolution of the poloidal magnetic field perturbation, $\delta {B}_{\theta }$, at toroidal angle *ϕ* of 90°, 198°, and 270° in a toroidal array^20^, displacement of the contour of electron temperature at ${r}_{{\rm{e}}ff}/{a}_{99}$ = 0.77, and RF intensity measured with an RF radiation probe^21^ at a toroidal angle *ϕ* of 121°. The effective radius of the location of the magnetic probe array is ${r}_{{\rm{e}}ff}/{a}_{99}$ = 1.3–1.4^22^. The behavior of the magnetic field perturbation is quite different depending on the toroidal angle, as seen in Fig. 1(a–c). Here the amplitude of the poloidal magnetic field is derived by integrating the probe signal with a high-pass filter (>1 kHz) in order to eliminate the drift of the signal. While the MHD burst, which is characterized by the large amplitude of magnetic field oscillations appears in all magnetic probes, out of phase oscillations appear in the magnetic probes at the toroidal angles of 90 and 270 degrees, respectively, and no oscillation appears at the toroidal angle of 198 degree between bursts. This indicates that the MHD mode between the MHD bursts are $m/n$ = 1/1 stationary (non-rotating) modes, while the MHD bursts are rotating modes. Figure 1(d) shows the amplitude of the *n* = 1 *m* = −1 (clockwise direction, CW) component of this stationary MHD mode. This amplitude of the stationary MHD mode shows several series of growth and decay (3 $\sim$ 13 times) between MHD bursts. It should be noted that there is no increase in the amplitude of the last stationary MHD mode of the series (just before the MHD bursts). This indicates that the stationary MHD mode does not trigger MHD bursts.

The displacement of the plasma, ${\xi }_{Te}$, is evaluated from the fast change (1–10 kHz) in temperature, $\delta {T}_{e}$, and a quasi-stationary (<40 Hz) temperature gradient measured with electron cyclotron emission (ECE)^23^ at the toroidal angle *ϕ* of 198° defined as $\delta {T}_{e}$/$\nabla {T}_{e}$. As seen in Fig. 1(e), large displacements of plasma at ${r}_{{\rm{e}}ff}/{a}_{99}$ = 0.77 exceed 1.5 cm at the beginning of each MHD burst. Here the positive displacement is an outward shift and negative displacement is an inward shift. It is interesting that these displacements are most significant at the non-rational surface of ${r}_{{\rm{e}}ff}/{a}_{99}$ = 0.77, which is much deeper inside the *q* = 1 rational surface located at ${r}_{{\rm{e}}ff}/{a}_{99}\sim 0.89$. The sharp increase of the RF signal indicates the timing of the collapse of energetic ions as seen in Fig. 1(f).

### Stationary MHD mode at rational surface and tongue-shaped deformation at non-rational surface

**Figure 2:**
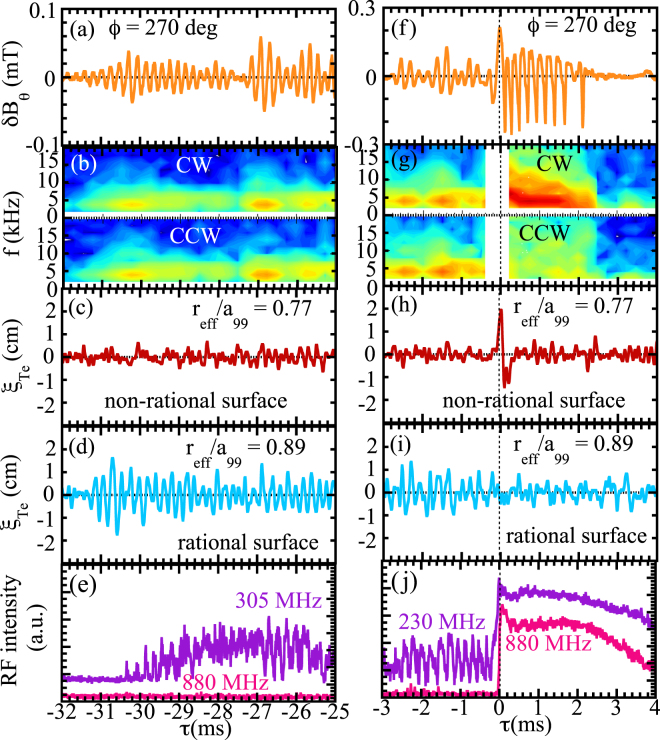
Time evolution of (**a**,**f**) poloidal magnetic field perturbation, $\delta {B}_{\theta }$, at toroidal angle *ϕ* of 270°, (**b**,**g**) contour of amplitude of *m* = −1 (CW) and *m* = 1 (CCW) component of $\delta {B}_{\theta }$, displacement of the contour of electron temperature at (**c**,**h**) non-rational surface (${r}_{{\rm{e}}ff}/{a}_{99}$ = 0.77), (**d**,**i**) rational surface (${r}_{{\rm{e}}ff}/{a}_{99}$ = 0.89), (**e**,**j**) RF intensity of low frequency (230, 305 MHz) and high frequency (880 MHz) at (**a**–**e**) the onset of stationary mode and (**f**–**j**) the tongue event. Here *τ* is the relative time with respect to the collapse of energetic ions as indicated by the sharp increase in RF intensity at *t* = 4.62630 (second spike of RF intensity in Fig. 1).

Figure 2 shows the expanded view of the magnetic field probe signals, displacement of the contour of electron temperature and the RF signal at the onset of the MHD mode and the onset of the tongue-shaped deformation. Here *τ* is a relative time with respect to the abrupt sharp increase in RF intensity. As seen in Fig. 2(a,f), the MHD mode starts to grow immediately after the previous MHD burst (∼4 ms) and well before (∼−32 ms) the next MHD burst, and repeats spontaneous growth and decay in the time scale of 0.5 ∼2 ms. The contour of the frequency of $\delta {B}_{\theta }$ in Fig. 2(b,g) shows that this MHD mode has the same amplitude for the CW and the counter clockwise (CCW) components, which indicates that this is a stationary (non-rotating) mode, while the MHD mode after the tongue event is a rotating mode in the CW direction. The amplitude of the magnetic field perturbation becomes larger and the growth/decay time becomes shorter and the displacement is localized near the rational surface. The large displacement abruptly appears at the non-rational surface just before the collapse of energetic ions (*τ* = 0) which is a clear indication of the tongue-shaped deformation as seen in Fig. 2(c,h). After the collapse of energetic ions, large oscillations of magnetic field are observed in all magnetic probes. The rotation frequency of the mode decreases in time and the mode rotation abruptly stops 2 ms later.

The displacement at the rational surface becomes even smaller during the MHD burst, while the amplitudes of the magnetic field perturbations are large, as seen in Fig. 2(d,i). RF signals with low frequencies of 230 MHz and 305 MHz show amplitude oscillations with the same frequency as that of magnetic field and of the displacement of the contour of electron temperature at a rational surface. This oscillation indicates the loss of energetic ions, which has a resonance to mode frequency of MHD burst. In contrast, the sharp increase and gradual decay of the RF intensity is observed in the high frequency of 880 MHz only at the tongue event. This sharp increase indicates the abrupt large loss (collapse) of energetic ions, which is different from the resonant loss observed at low frequency.

### Causality relation between the MHD burst and collapse of energetic ions

**Figure 3:**
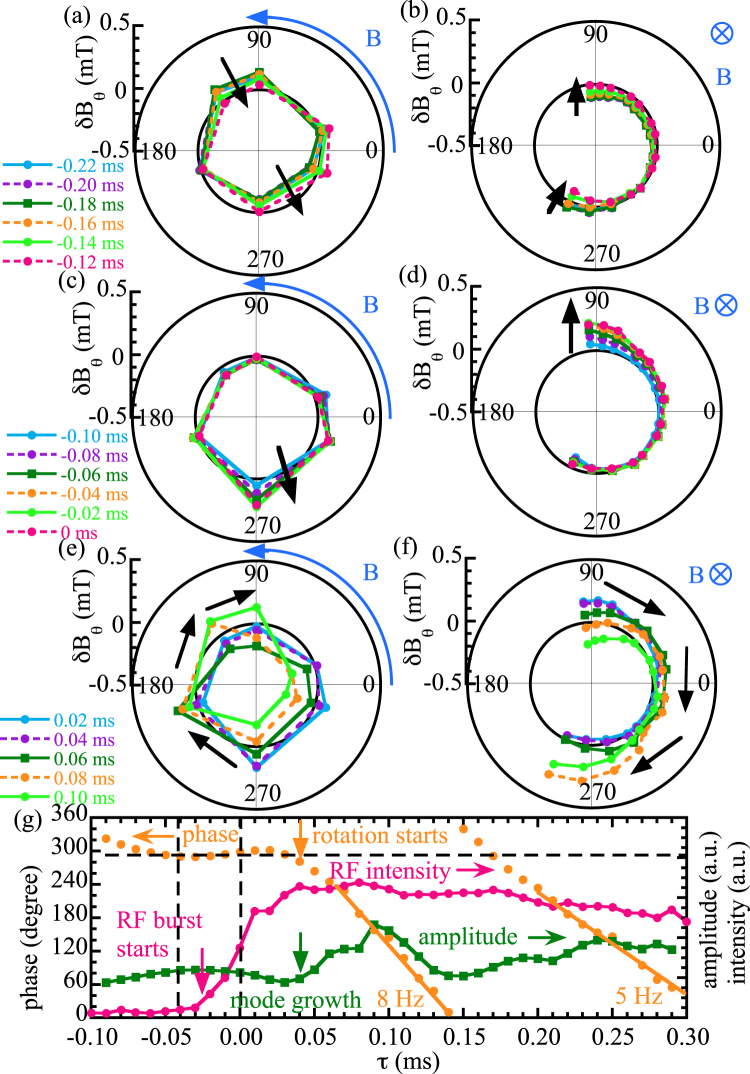
Polar plot of poloidal magnetic field perturbation, $\delta {B}_{\theta }$, of (**a**,**c**,**e**) toroidal array and (**b**,**d**,**f**) poloidal array at (**a**,**b**) the end of stationary *m*/*n* = 1/1 MHD mode, (**c**,**d**) the tongue-shaped formation, and (**e**,**f**) the start of a rotating *m*/*n* = 1/1 MHD mode, and (**g**) time evolution of phase and amplitude of a 1/1 mode of $\delta {B}_{\theta }$ and 880 MHz RF intensity. Here the legends show the relative time of *τ* in Fig. 2.

The different characteristics of the three phases of the MHD activity are clearly observed in the toroidal and poloidal structures of magnetic field perturbations and radial profiles of displacement. Figure 3 shows the toroidal and poloidal distributions of the perturbation of poloidal magnetic field measured with probe arrays during the stationary *m*/*n* = 1/1 MHD mode, tongue-shaped deformation and rotating *m*/*n* = 1/1 MHD mode. The toroidal and poloidal distribution of the poloidal magnetic field perturbation, $\delta {B}_{\theta }$, of the quasi-stable mode, starting well before the collapse of energetic ions (*τ* = −31 ms) and ending just before the tongue-shaped formation (*τ* = −0.1 ms), has clear stationary 1/1 MHD mode patterns where the perturbations at 180 degrees apart are out of phase. The perturbations of the stationary 1/1 MHD are largest at the port with the toroidal angles of 90 and 306 degrees where the perpendicular NBIs are injected This fact implies that the energetic particle perpendicular to the magnetic field is responsible for causing this MHD instability.

In contrast, there is no 1/1 mode structure observed in the toroidal and poloidal distributions of the poloidal magnetic field perturbation, $\delta {B}_{\theta }$, at the onset of the tongue event of −0.1 ms to 0 ms in relative time. The perturbation is localized at 270 degree in toroidal angle near the port where the perpendicular NBI is injected and at 90 degrees in poloidal angle. These results suggest that the pressure gradient of the trapped ion provided by the NBI is the most likely candidate for causing this tongue-shaped deformation. The tongue-shaped deformation becomes maximum just before the collapse of energetic ions indicated by the abrupt sharp increase in RF intensity. The 1/1 MHD mode starts to rotate at 0.04 ms after the collapse of energetic ions in the clockwise direction in both toroidal and poloidal directions with a frequency of $\sim$8 kHz, which is consistent with the resonant frequency of helically trapped ions from the perpendicular NBI^13^. The rotation frequency of the mode decreases from 8 kHz to 5 kHz within one cycle as seen in Fig. 3(g). The 1/1 mode amplitude starts to increase when the mode rotation starts, which suggests that the mode growth is associated with the mode rotations. However, the initial start of the rotating 1/1 MHD mode always occurs after (but not before) the collapse of energetic ions.

### Magnetic field reconnection and parity change of resonant MHD mode

**Figure 4:**
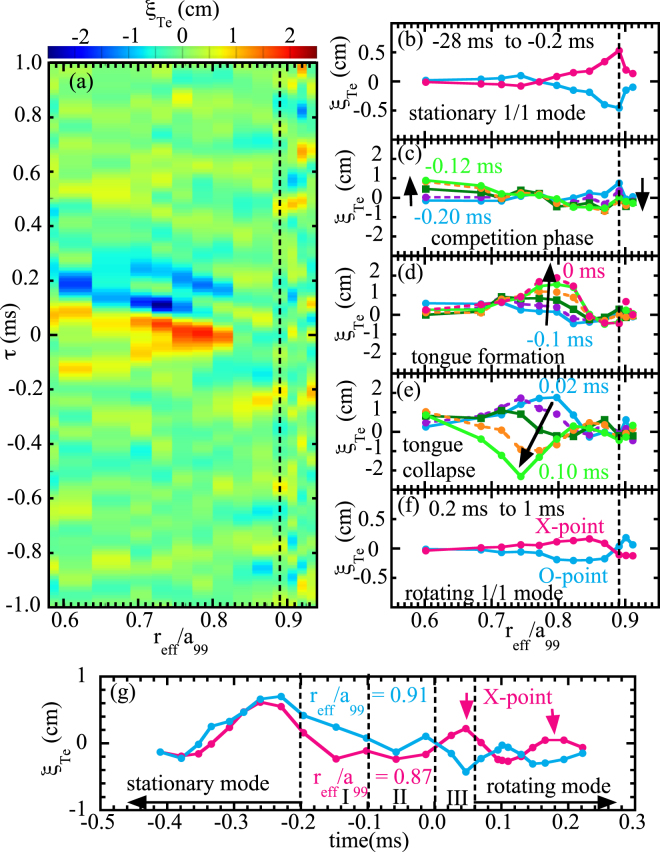
(**a**) Contour of plasma displacement in space and time and radial profiles of the plasma displacement in phase of (**b**) stationary 1/1 MHD mode, (**c**) competition of 1/1 mode and tongue, (**d**) tongue formation, (**e**) tongue collapse, (**f**) rotating 1/1 MHD mode afterwards, and (**g**) time evolution of plasma displacement at ${r}_{{\rm{e}}ff}/{a}_{99}$ = 0.87 and 0.91 across the rational surface.

Figure 4 shows the contour of displacement at the tongue event and the radial profiles of displacement at different phases. As seen in the contour plot, the plasma displacement of the tongue event starts well inside the rational surface (${r}_{{\rm{e}}ff}/{a}_{99}$ = 0.6) and the peak displacement moves outwards during the tongue-shaped formation. Then the peak displacement moves inward during the tongue collapse, which is typically observed during the tongue event. It should be noted that the tongue event lasts only 100 *μ*s and can not be identified with conventional mode analysis, where a time window longer than one period of oscillation is necessary (see Fig. 3(d) in^13^). In the stationary 1/1 MHD mode phase for −28 ms to −0.2 ms in relative time, the displacement is 6 mm and localized at ${r}_{{\rm{e}}ff}/{a}_{99}$ = 0.89 near the location of the rational surface of *q* = 1. It should be noted that the displacement has interchange parity (in-phase across the rational surface). Just before the tongue event for −0.20 to −0.12 ms in relative time, the displacement near the rational surface decreases and the displacement much deeper inside ${r}_{{\rm{e}}ff}/{a}_{99}$ = 0.6 starts to increase. At the tongue formation period for −0.1 ms to 0 ms in relative time, a significant displacement appears well inside the *q* = 1 rational surface. The displacement develops outward and reaches the value of 20 mm before the collapse. This displacement of the plasma is much larger than the displacement (∼3 mm) at ${r}_{{\rm{e}}ff}/{a}_{99}$ = 0.77 evaluated from the amplitude of the 1/1 mode component plotted in Fig. 3(g).

Associated with the collapse of energetic ions, this tongue collapses from 0.02 ms to 0.10 ms in relative time as seen in Fig. 4(e). The displacements during the rotating 1/1 MHD mode after the tongue event are relatively small (less than 3 mm) and show the oscillation between the X-point structure (positive inside negative outside) and the O-point structure (negative inside positive outside) as seen in Fig. 4(f), although the perturbation of the poloidal magnetic field is large. Here, in order to improve the signal to noise ratio, the conditioning average with the phase of the magnetic field perturbation is applied from −28.0 to −0.2 ms in relative time for the stationary 1/1 MHD mode and from 0.2 to 1.0 ms in relative time for the rotating 1/1 mode in 32 MHD bursts (4.2–5.0 sec). In Fig. 4(b,f), the positive and negative maximum displacement are plotted. The rotating 1/1 MHD mode has tearing parity (out-of-phase across the rational surface) which is in contrast to the interchange parity of the stationary 1/1 mode before the tongue event. As seen in the time evolution of the displacement on the inner side and the outer side of the rational surface of ${r}_{{\rm{e}}ff}/{a}_{99}$ = 0.89 in Fig. 4(g), the displacements at the onset of this rotating mode have a X-point structure.

### Discussion and Summary

The most important finding in this experiment is that there are three states: 1) stationary 1/1 mode with interchange parity, 2) tongue-shaped deformation at non-rational surface, and 3) rotating 1/1 mode with tearing parity in the discharge with MHD bursts. When the tongue-shaped deformation occurs, it curbs the *m*/*n* = 1/1 mode by taking the free energy of the instability due to the sharp gradient of energetic ions and triggers a collapse of energetic ion, which causes the plasma rotation and MHD burst. The tongue-shaped deformation triggers the collapse of energetic ions detected by the sharp increase in RF intensity. The MHD burst, characterized by the rotating m/n = 1/1 mode, starts ~40 $\mu$s after (not before) the collapse of energetic ions, which shows that the MHD burst is not the cause but the result of the collapse of the energetic ions. The stationary *m*/*n* = 1/1 mode, which is excited at the rational surface by the large perpendicular beam pressure gradient in the plasma, does not trigger the collapse of energetic ions because the frequency of mode rotation does not match the precession frequency of helically trapped energetic ions.

The magnetic topology change in the MHD mode from interchange parity to tearing parity observed in this experiment is quite interesting. The positive slope of the displacement across the rational surface at the O-point phase (${\xi }_{Te}$ = −17 mm at *R* = 4347 mm and ${\xi }_{Te}$ = +18 mm at *R* = 4384 mm) in Fig. 4(f) shows the flattening of the temperature inside the magnetic island. These results clearly demonstrate that the tongue-shaped deformation triggers the magnetic field reconnection and formation of the magnetic island. As seen in Fig. 3(g), the growth time of the magnetic island is of the order of a few tens of microseconds and is much faster than the resistive reconnection timescale. In the present situation, the tongue-shaped deformation is localized in the toroidal direction. Thus the reconnection might occur highly inhomogeneously along the toroidal direction. Acceleration of the reconnection by the complex geometry of the return current has been predicted^24^. This experiment and another similar report^25^ strongly stimulate study on the interaction between non-resonant/non-linear perturbations and ordinary resonant MHD modes.

## Methods

### Large Helical Device

The Large Helical Device (LHD) is a heliotron type device for the magnetic confinement of high temperature plasmas with the magnetic field, B, of 2.7 T at the magnetic axis in the vacuum field, major, *R*_*ax*_, and effective minor radius ${r}_{{\rm{e}}ff}$, of 3.6 m and 60–65 cm, respectively. In this experiment, the plasma density is $1-2\times {10}^{19}$ m^−3^ and the central temperature is in the range of 2–4 keV. The LHD is equipped with three tangential neutral beams (NBs) in opposite injection directions (two CCW and one CW) and two perpendicular neutral beams.

### Magnetic probes

Magnetic probes using advanced technology are installed inside the vacuum vessel of the LHD device to measure the perturbation of the poloidal magnetic field at 6 toroidal locations (*ϕ* = 18°, 90°, 126°, 198, 270°, 342°) and 14 poloidal locations ($\theta$ = 245°, 264°, 285°, 306°, 316°, 338°, 349°, 11°, 22°, 44°, 54°, 75°, 86°, 96°). The sensitivity is absolutely calibrated and the effective radius of the location of the magnetic probe array is ${r}_{p}$ = 1.35. The poloidal magnetic field perturbation, $\delta {B}_{\theta }$, in the plasma decays in radius. The displacement of the magnetic flux surface, $\xi$, can be evaluated from $\delta {B}_{\theta }$ measured as $c{({r}_{p}-{r}_{\xi })}^{n+1}\delta {B}_{\theta }$, where ${r}_{\xi }$ is the effective radius of the displacement, *n* is the toroidal mode number of the MHD mode, and *c* is a constant.

### RF radiation probe

Cyclotron motions of energetic ions in a plasma radiate electromagnetic waves in a frequency range from tens to hundreds of MHz. The RF radiation probe on LHD consists of a dipole antenna in the vacuum vessel and spectrometers. The dipole antenna is located at the midplane of the vacuum vessel of the LHD device ($\theta$ ∼ 0°). The filter-bank resolves the RF intensity signal into 8 frequency components of 185, 230, 305, 410, 600, 880, 1130, and 1630 MHz. The intensity of RF radiation probes is widely used as a timing indicator for the energetic ion loss from the plasma, because the RF probe has a high time resolution and is sensitive to the high frequency RF signals excited by the loss of energetic ions at the plasma edge.

### Electron cyclotron emission (ECE)

Electron cyclotron emission (ECE) intensity emitted from the plasma is proportional to the electron temperature (*T*_*e*_) and ECE is widely used for a diagnostic of plasma electron temperature. Because the change in the electron temperature due to the heating is relatively slow and on the order of the confinement time scale (>10–100 ms), the fast change in *T*_*e*_ represents the displacement of the equi-temperature surface. Therefore, the displacement of the equi-temperature surface, ${\xi }_{Te}$, is evaluated from the fast change (1–10 kHz) in temperature $\delta {T}_{e}$ and quasi-stationary (<40 Hz) temperature gradient measured with ECE as $\delta {T}_{e}$/$\nabla {T}_{e}$. In this article, the displacement of the equi-temperature surface (called plasma displacement) is indicated as ${\xi }_{Te}$ in order to distinguish from the displacement of the magnetic flux surface, $\xi$. When the plasma is frozen into the magnetic field, which is the case for typical MHD modes, ${\xi }_{Te}$ = $\xi$.

### Conditional reconstruction

Conditional reconstructions of data are used to improve the time resolution of the measurements when there are many repeated events. By taking the relative time difference of the measurements with respect to the events, the effective time resolution can be improved when several events randomly occur in steady state in the discharge.

## References

[1] Itoh S (1998). Physics of collapse events in toroidal plasmas. Plasma Phys. Control. Fusion.

[2] Wesson JA (1991). Spontaneous m = 1 instability in the JET sawtooth collapse. Nucl. Fusion.

[3] Campbell, D. *et al*. JET Team (presented by Campbell, D. J.) 1991 Plasma Physics and Controlled Nuclear Fusion Research 1990 vol 1 (Vienna: IAEA) p 437.

[4] Wesson, J.A., Tokamaks 4th edition, Oxford Univ. Press (Oxford, 2011).

[5] Kusano K (2004). The trigger mechanism of solar flares in a coronal arcade with reversed magnetic shear. ApJ.

[6] Kusano K (2012). Magnetic field structures triggering solar flares and coronal mass ejections. ApJ.

[7] Yun GS (2011). Two-Dimensional Visualization of Growth and Burst of the Edge-Localized Filaments in KSTAR H-Mode Plasmas. Phys. Rev. Lett..

[8] Yun GS (2012). Two-dimensional imaging of edge-localized modes in KSTAR plasmas unperturbed and perturbed by n = 1 external magnetic fields. Phys. Plasmas.

[9] Toi K (2000). Energetic ion driven MHD instabilities observed in the heliotron/torsatron devices Compact Helical System and Large Helical Device. Nucl. Fusion.

[10] Yamamoto S (2005). Experimental studies of energetic-ion-driven MHD instabilities in Large Helical Device plasmas. Nucl. Fusion.

[11] Nagaoka K (2008). Radial Transport Characteristics of Fast Ions Due to Energetic-Particle Modes inside the Last Closed-Flux Surface in the Compact Helical System. Phys. Rev. Lett..

[12] Ogawa K (2010). Observation of energetic-ion losses induced by various MHD instabilities in the Large Helical Device (LHD). Nucl. Fusion.

[13] Du XD (2015). Resistive Interchange Modes Destabilized by Helically Trapped Energetic Ions in a Helical Plasma. Phys. Rev. Lett..

[14] Heidbrink WW (2011). Characterization of off-axis fishbones. Plasma Phys. Control. Fusion..

[15] Schild P, Cottrell GA, Dendy RO (1989). Sawtooth oscillations in ion cyclotron emission from JET. Nucl Fusion.

[16] Dendy RO (1994). The excitation of obliquely propagating fast Alfvén waves at fusion ion cyclotron harmonics. Phys. Plasmas.

[17] Arstimovich, L.A. A Physicist’s ABC onPlasma, First edition 1978, Revised from the 1976 Russian Editions, English translation, Mir Publishers (Moscow, 1978).

[18] Itoh K (2016). On Magnetic Signals of a Large-Scale Quasi-electrostatic Perturbation. J. Phys. Soc. Jpn..

[19] Ida K (2016). Abrupt onset of tongue deformation and phase space response of ions in magnetically confined plasmas. Sci. Rep..

[20] Sakakibara S (2010). Magnetic measurements in LHD. Fusion Sci. Technol..

[21] Saito K (2013). Measurement of Ion Cyclotron Emissions by Using High-Frequency Magnetic Probes in the LHD Plasma Science and Technology.

[22] Sakakibara S (2001). MHD characteristics in the high beta regime of the Large Helical Device. Nucl. Fusion.

[23] Nagayama Y (1999). Electron cyclotron emission diagnostics on the large helical device. Rev. Sci. Instrum..

[24] Che H, Drake JF, Swisdak M (2011). A current filamentation mechanism for breaking magnetic field lines during reconnection. Nature.

[25] Lee JE (2017). Solitary perturbations in the steep boundary of magnetized toroidal plasma. Sci. Rep..

